# Next-generation sequencing for MRD monitoring in B-lineage malignancies: from bench to bedside

**DOI:** 10.1186/s40164-022-00300-2

**Published:** 2022-09-03

**Authors:** Xinyue Deng, Meilan Zhang, Jianfeng Zhou, Min Xiao

**Affiliations:** 1 Department of Hematology, Tongji Hospital, Tongji Medical College, Huazhong University of Science and Technology, Wuhan, Hubei 430030 China; 2 Immunotherapy Research Center for Hematologic Diseases of Hubei Province, Wuhan, Hubei 430030 China

**Keywords:** High-throughput sequencing, V(D)J recombination, Hematological malignancies, Minimal residual disease, Chimeric antigen receptor

## Abstract

Minimal residual disease (MRD) is considered the strongest relevant predictor of prognosis and an effective decision-making factor during the treatment of hematological malignancies. Remarkable breakthroughs brought about by new strategies, such as epigenetic therapy and chimeric antigen receptor-T (CAR-T) therapy, have led to considerably deeper responses in patients than ever, which presents difficulties with the widely applied gold-standard techniques of MRD monitoring. Urgent demands for novel approaches that are ultrasensitive and provide sufficient information have put a spotlight on high-throughput technologies. Recently, advances in methodology, represented by next-generation sequencing (NGS)-based clonality assays, have proven robust and suggestive in numerous high-quality studies and have been recommended by some international expert groups as disease-monitoring modalities. This review demonstrates the applicability of NGS-based clonality assessment for MRD monitoring of B-cell malignancies by summarizing the oncogenesis of neoplasms and the corresponding status of immunoglobulin (IG) rearrangements. Furthermore, we focused on the performance of NGS-based assays compared with conventional approaches and the interpretation of results, revealing directions for improvement and prospects in clinical practice.

**Supplementary Information:**

The online version contains supplementary material available at 10.1186/s40164-022-00300-2.

## Introduction

The minimal residual disease (MRD) level achieved at the end of induction/consolidation therapy is a recognized important factor for risk stratification and relapse prediction in several hematological malignancies, such as acute lymphoblastic leukemia (ALL) [1, 2], multiple myeloma (MM) [3, 4], mantle cell lymphoma (MCL) [5, 6], follicular lymphoma (FL) [7], diffuse large B-cell lymphoma (DLBCL) [8], and chronic lymphocytic leukemia (CLL) [9]. Significantly better recovery and survival outcomes in patients with negative MRD at certain time points were observed in multiple studies [10–12]. Nevertheless, relapses among patients with negative MRD confirmed by conventional methods occur, especially among patients treated with novel strategies such as CAR-T therapies and lack preliminary MRD data [13], indicating the importance of assessing the required depth, ideal test sensitivity, and proper definition of MRD negativity. MRD negativity is usually defined as less than 1 tumor cell in 100,000 bone marrow (BM) cells (1 × 10^–5^); however, recent evidence suggests that prolonged progression-free survival (PFS) and/or overall survival (OS) outcomes are observed when the threshold for MRD negativity is changed to 1 × 10^–6^ [14].

The traditional, widely applied methods used for MRD monitoring mainly include polymerase chain reaction (PCR)-based strategies (e.g., allele-specific oligonucleotide-real time quantitative PCR, ASO-RQ-PCR) and immunophenotype-based strategies (e.g., multicolor flow cytometry, MFC) with sensitivities of approximately 1 × 10^–5^ [11] and 1 × 10^–4^ [15], respectively. Imaging techniques (e.g., positron emission tomography-computed tomography, PET-CT) are generally less sensitive than MRD-based approach [16]. The advent of next-generation flow cytometry (NGF), which outperforms conventional MFC by the optimized combination of fluorochromes and antibody reagents, further improves the sensitivity of MRD detection to 2 × 10^–6^ [17]. Another alternative approach developed on the basis of clonality assessment through IG V(D)J rearrangements, NGS, emerged in parallel, exhibiting even higher sensitivity up to 1 × 10^–6^ and providing more substantial genetic-level information [18, 19].

Compared with other MRD monitoring methods, the NGS-based IG clonality approach possesses substantial potential for a wide range of applications due to the distinct features of the technique, such as the comprehensiveness of the information gained, the ability to recognize clonal evolution, and the ability to standardize the workflow [20–23]. Furthermore, NGS-based is recommended in the NCCN Guidelines for MRD monitoring for ALL, CLL, and MM, indicating considerable market prospects. However, the relatively insufficient data from clinical trials and the lack of systematic summaries on the working principles and the scope of application have seriously impeded the promotion of IG NGS-based MRD monitoring. This review demonstrates the feasibility and reasonability of the IG NGS approach applied for MRD detection of B-cell malignancies from multiple perspectives, including the distinct features of IG rearrangements in different neoplasms and the interpretation of corresponding IG NGS results, a comparison of the performance of present MRD methods and the unique advantages of NGS-based methods, and, most importantly, a summary of the current clinical studies involving NGS-based MRD monitoring, highlighting translational medicine applicability and the use of high-throughput technology in clinical practice.

## The rearrangement of Ig genes in normal B cells

During the process of maturation, the immunoglobulin genes in normal B cells undergo a process referred to as V(D)J recombination to produce a unique receptor (B-cell receptor, BCR) for combination with its specific antigen (Additional file 1 Fig. S1). At chromosome 14 (14q32.33), the IGHV, IGHD, and IGHJ segments of the immunoglobulin heavy chain (IGH) gene are rearranged in an ordered fashion, while at chromosome 2 (2p11.2), IGKV and IGKJ or IGLV and IGLJ (chromosome 22:22q11) undergo the same phenomenon but at a later time [24]. The whole process is completed through by precisely controlled enzymatic machinery mediated by the interaction of rag (coded by recombination activating gene, RAG) with recombination signal sequence (RSS) motifs located near the V(D)J segments and the subsequent splice site [25]. During the pro-B-cell stage, D to JH recombination precedes VH to DJH with deletion of the intervening gene segments, ultimately producing an intact and unique variable (V) region. The rearrangement events that occur at the light chain locus are much more sophisticated, involving both deletions and inversions due to the participation of IGKV(D) clusters, which are located upstream of IGKV clusters and have similar sequences and opposite-orientation RSSs [26–28].

**Fig. 1:**
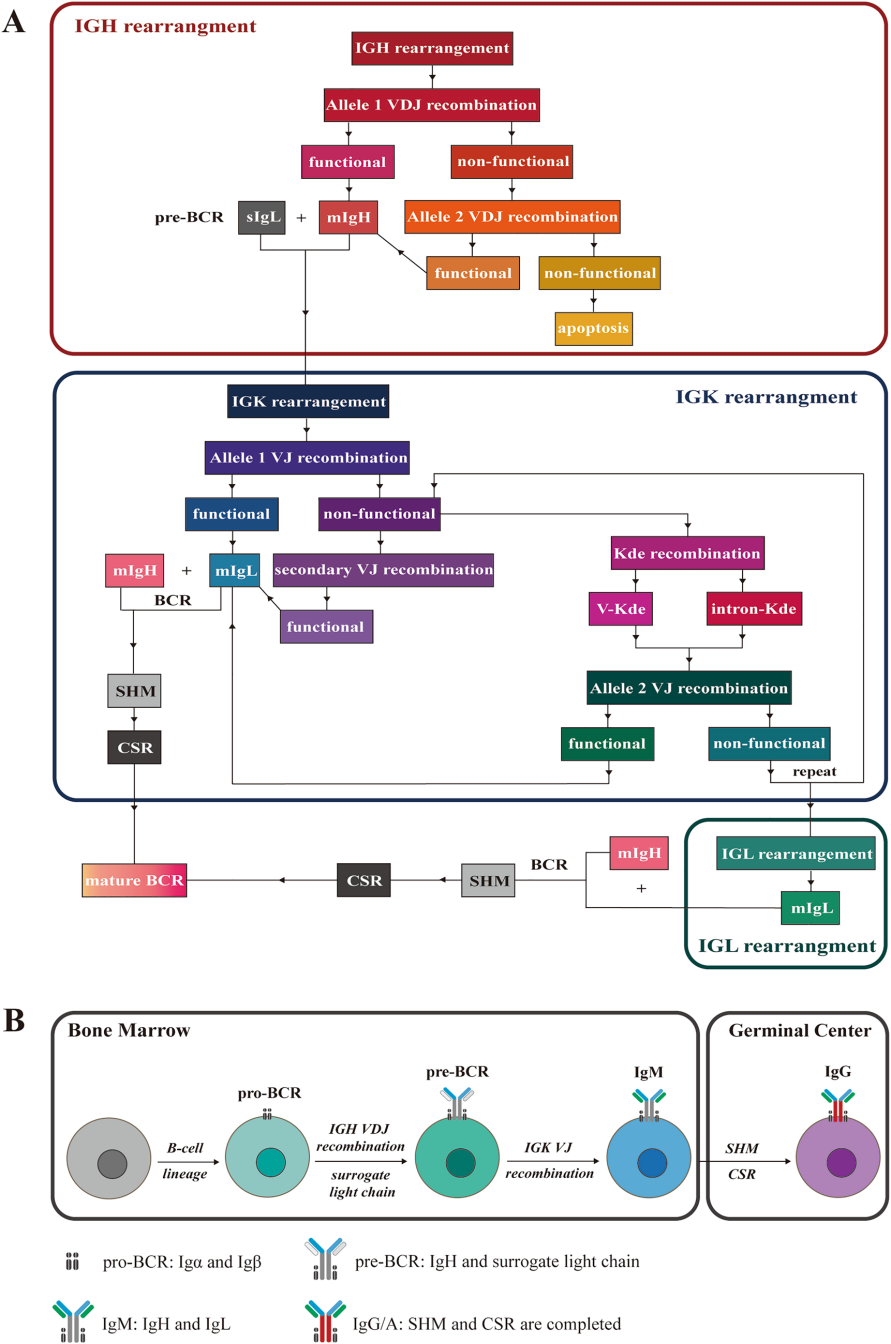
Summary of the allelic exclusion theory and the normal B cell differentiation process. **A** Heavy chain rearrangement precedes light chain rearrangement, and recombination of the IGK segment precedes IGL. SHM and CSR occur in GC after successful Ig rearrangement to produce mature BCR. **B** Maturation of B cells from hematopoietic stem cells to mature B cells with class-switched BCR (IgA/IgG) through Ig rearrangement and BCR signaling

Based on previous studies [29], the principles of V(D)J rearrangements, also designated allelic exclusion, were analyzed and summarized (Fig. 1). The rules are implemented as follows: (1) the IGH gene segments must rearrange to produce a functional V region that can successfully pass the in-frame selection. (2) The nonproductive IGH rearrangements are inactivated in parallel with the initiation of the second rearrangement at another allele. (3) Rearrangements of IGK gene segments occur after the appropriate IGH rearrangement, while failed IGH rearrangements occurring in both alleles result in apoptosis. (4) A second rearrangement can occur in either allele when the first IGK fails to produce a functional result. (5) The nonproductive IGK rearrangements are inactivated by the deletion of intervening DNA sequences through either Kde-IGKJ or intron-Kde recombination [30]. (6) Rearrangements of IGL gene segments occur after failed IGK rearrangements in both alleles, while productive IGK recombination tends to leave the IGL gene in its germline configuration [31, 32]. The successful expression of IgH marks the transition from pro-B cells to pre-B cells, in which IgH combines with a surrogate light chain to form a pre-BCR, activating in-frame selection events and light chain rearrangements. Following somatic hypermutation (SHM) and class-switch recombination (CSR) in the germinal center (GC), immature B cells from the BM are converted into mature B cells equipped with antigen specific, high-affinity, and unique BCRs [33] and then differentiate into either plasma cells (PCs) or memory B cells [34, 35] (Fig. 1).

## Targets utilized for MRD monitoring by NGS

The high diversity of the BCR repertoire results from nearly infinite combinations of V(D)J gene segments. The 123–129 VH gene segments, of which 38–46 segments are functional and 36 are considered pseudogenes, can be approximately grouped into 7 families (VH1–VH7) [36]. A total of 27 D gene segments and 9 JH segments comprise 23 and 6 functional components, respectively [37]. The possible VH/D/JH combinations and SHM that occur in hotspots endow the complementarity determining regions 3 (CDR3) of BCRs with the ability to interact with distinct antigen epitopes and serve as a fingerprint when recognizing and tracking the specific B-cell [12]. Similar processes occur at the IGK or IGL locus, within which 34–38 and 29–33 functional segments can be selected from a total of 76 Vκ and 73–74 Vλ genes, respectively [38]. Recombination in the constant region of the IG gene initiates during CSR in the GC environment [39]. By simply deleting the intervening DNA sequence between the S region of Cμ and another constant component, the IgM isotype of BCR is converted into a mature IgA or IgG isotype [39].

During adaptive immune responses, B cells react to antigenic stimulation and rapidly proliferate, forming clones with the same V(D)J pattern and possible intraclonal diversity at the nucleotide level attributed to SHM. Similarly, after malignant transformation at a certain time point during B cell differentiation triggered by either activation of oncogenes or inactivation of tumor suppressor genes, B-lineage cancer cells carrying the same complete V(D)J or incomplete DJ rearrangement unlimitedly multiply with possible subclone characteristics caused by ongoing SHM or ongoing V(D)J recombination, respectively [40–42]. Given that the rearranged IG gene is unique and the quantification of these specific sequences will dramatically increase to a level far beyond the background of the normal IG gene repertoire when malignant cells proliferate, it is convincing and feasible to consider the IG V(D)J rearrangement pattern as an alternative for both clonality assessment of diagnosis and targets of MRD monitoring.

## The oncogenesis of B-lineage malignancies and the corresponding status of IG rearrangements

**Fig. 2:**
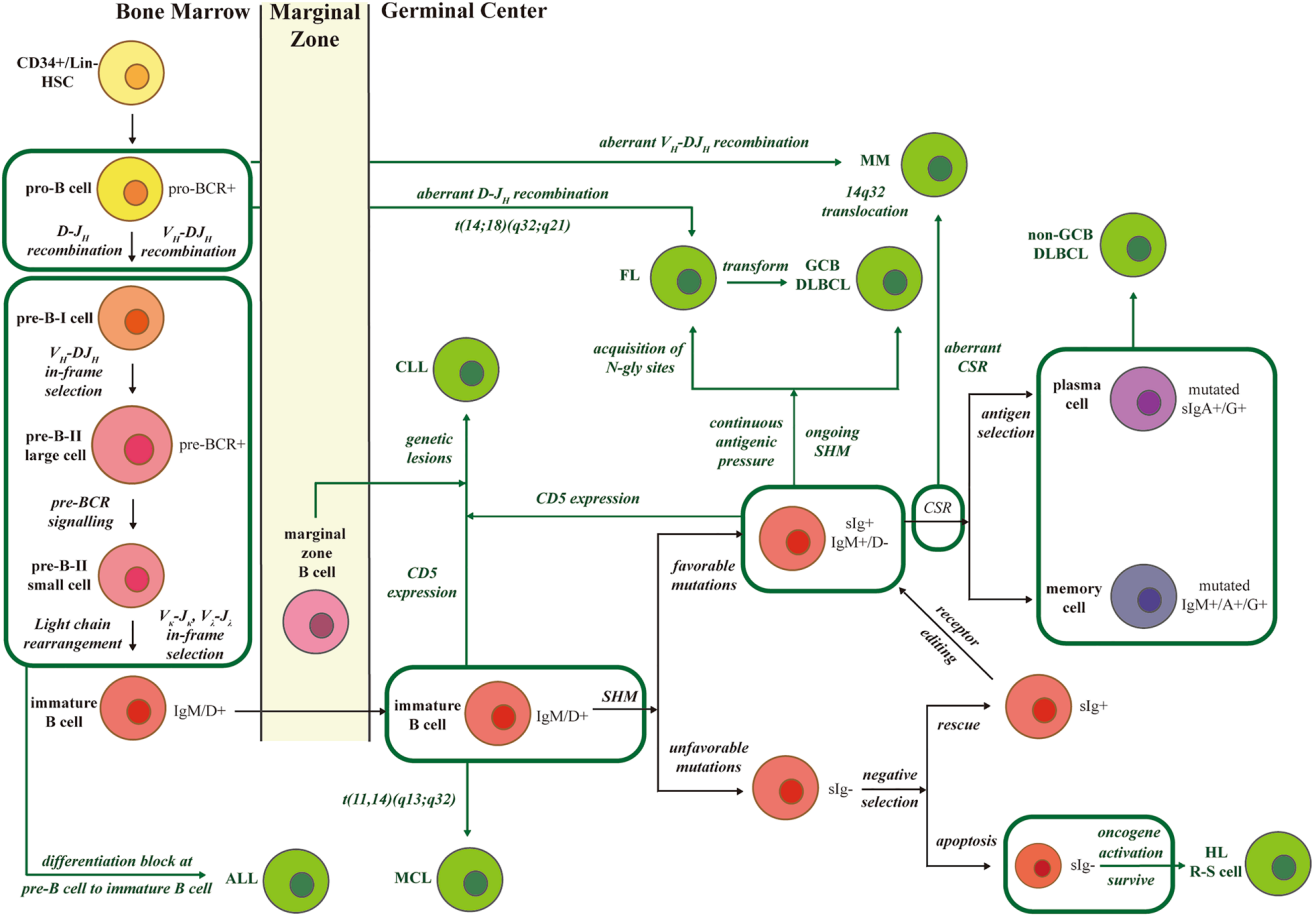
Schematic representation of the oncogenesis of B-lineage malignancies. The t(14;18)(q32;q21) rearrangement caused by aberrant D-JH recombination during the pro-B-cell stage plus the acquisition of N-gly sites during the SHM period ultimately leads to FL. The blockade at the pre-B-cell stage to the immature B-cell stage in parallel with the ongoing recombination events promotes the development of ALL. MCL originates from immature B cells with t(11;14)(q13;q32). GCB-DLBCL is transformed from B cells under continuing antigenic pressures in GC characterized by ongoing SHM or is transformed from FL, while the non-GCB subtype originates from plasma cells or memory-like B cells that have completed the GC reaction. MM is caused by an aberrant translocation involving the IGH locus (14q32), which occurs during V(D)J recombination, SHM or CSR. HL derives from surviving cells that escape from apoptosis caused by unfavorable mutations by the activation of oncogenes. N-gly sites, asparagine-X-serine/threonine sites

The deletion–recombination reactions of V(D)J rearrangement, SHM, and CSR require a double-strand break (DSB) at a specific locus, introducing potential aberrant translocation events that can serve as distinguishing signs in fluorescence in situ hybridization or IG-based clonality assessment [43–46]. Characteristics associated with the abnormal IG repertoire, such as biased V-J usage [40, 47], stereotyped CDR3 [48–52], the tendency to mutate frequently [18, 45, 53] or retain germline configuration [40, 54, 55], ongoing SHM [56–58] or lack of intraclonal diversification [46], were further confirmed in many hematology studies based on the sequencing of IG genes. Detailed information is exhibited in Table 1 and Fig. 2 to demonstrate the interpretation of sequence analysis of different B-linage malignancies.

**Table 1:** IGH/K rearrangements in different B-cell malignancies

B-cell malignancies	Origin	IG rearrangements			Clonal evolution	Citation
		V(D)J rearrangement	SHM and CSR	Other features		
ALL	Pre-B cells	IGH V-D-J usage: V_H_ usage: V_H_3 > V_H_1 > V_H_2, V_H_4 (most frequent: **V**_**H**_**6-1, V**_**H**_**1-2. V**_**H**_**3-11, V**_**H**_**3-13, V**_**H**_**3-15)** D usage: D2 > D3 > D6 (Most frequent: **D2-21** in pro-B ALL)J_H_ usage: J_H_4, J_H_6	Low-mutated or unmutated	High frequency of unproductive IGH rearrangements due to the continuously active recombinase enzyme	Continuing rearrangements process or independent new rearrangements	[41–43, 48, 60, 61, 94, 110]
		IGL V-J usage: V_κ_ usage: V_κ_1 > V_κ_2		No age-associated genotype pattern	Clonal selection during treatment	
				Various IG gene characteristics at diagnosis have no prognostic value	Oligoclonality of IGH at relapse is less frequent	
					Monoclonal IGH rearrangements/Major clones/clone with complete V-D-J recombination are stable	
MCL	Naïve mature B cells or memory-like B cells	IGH V-D-J usage: V_H_ usage: V_H_3 > V_H_4 > V_H_1 > V_H_2 (Most frequent: **V**_**H**_**3-21, V**_**H**_**3-23, V**_**H**_**4-34, V**_**H**_**1-8, V**_**H**_**4-59**) D usage: D3 > D6, D1 (Most frequent: **D3-22, D3-3**) J_H_ usage: J_H_4 > J_H_6	Minimally mutated or unmutated	Two molecular subtypes: conventional (cMCL) and leukemic non-nodal (nnMCL	Information unavailable	[45, 49–53, 55, 56, 62–65]
		IGL V-J usage: V_λ_ usage: V_λ_1, V_λ_2, V_λ_3 (Most frequent: **V**_**λ**_**2-14**) V_κ_ usage: Most frequent: **V**_**κ**_**3, V**_**κ**_**3-20**	t(11;14)q(13,32), the CCND1/IGH rearrangement	MCL express IgL-λ more frequently due to more K-de rearrangements		
		Stereotyped HCDR3 **V**_**H**_**4-34**/D2-2/J_H_6 **V**_**H**_**4-34**/D1-26/J_H_6 **V**_**H**_**3-21**/D3-9/J_H_6 **V**_**H**_**3-21**/D3-9/J_H_4 **V**_**H**_**3-21/**D6-6/J_H_6 + V_λ_3-19/J_λ_2	CCND2/CCND3 translocation with IGK/IGL	Highly restricted IG gene repertoire with stereotyped HCDR3 imply a role for antigen-driven selection in the oncogenesis		
		Stereotyped LCDR3: V_λ_3-19/J_λ_2-1 V_λ_2-14/J_λ_2-1 V_λ_2-14/J_λ_3-1 V_κ_3-10/J_κ_2-1 V_κ_3-10/J_κ_4-1				
CLL	B cells in GC	IGH V-D-J usage: V_H_ usage: V_H_3 > V_H_1, V_H_4 (Most frequent: **V**_**H**_**1-69, V**_**H**_**4-34, V**_**H**_**3-23, V**_**H**_**3-30, V**_**H**_**1-2**)	Differences of prognosis based on SHM level: Unmutated (U-CLL): SHM < 2%, pre-GC, worse survival Mutated (M-CLL): SHM > 2%, GC and post-GC, better survival	SHM in hotspots	Intra-clonal diversification within CLL is limited	[49–53, 68]
		Stereotyped HCDR3: **V**_**H**_**1-69**/D3-16/J_H_3 + V_κ_A27 **V**_**H**_**1-69**/D3-3/J_H_6 + V_λ_3-9 **V**_**H**_**3-21**/D3-3/J_H_6 + V_λ_2-14 **V**_**H**_**3-21** + V_λ_3-21 **V**_**H**_**4-34** + V_κ_2-30 **V**_**H**_**4-39**/D6-13/J_H_5 + V_κ_(D)1–39 **V**_**H**_**1-3**/D6-19/J_H_4 + V_κ_(D)1–39 **V**_**H**_**1-2**/D2-2/J_H_6 + V_κ_(D)1–39 **V**_**H**_**3-23**/D3-3/J_H_6 **V**_**H**_**3-23**/D4-23/J_H_3		Antigen selection		
				Stereotyped BCR, and most major subsets concerned unmutated with high conservation across the entire HCDR3		
				Satellite subsets to major subsets		
				Different ontogenetic trajectories for stereotyped and non-stereotyped cases		
				Autoreactive specificities		
**DLBCL**	B cells in GC	IGH V-D-J usage: V_H_ usage: V_H_1 > V_H_3 > V_H_4 > V_H_2 (Most frequent: **V**_**H**_**1-2, V**_**H**_**4-34, V**_**H**_**3-23, V**_**H**_**4-39, V**_**H**_**1-69, V**_**H**_**5-51, V**_**H**_**3-21**) D usage: D3, D2 (Most frequent: **D3-22, D3-10**) J_H_ usage: J_H_4, J_H_6	Ongoing SHM or mutated	Monoclonality is associated with poor prognostics	Two modes of clonally-related relapse: the early divergent mode and the late divergent mode	[49, 54, 57, 63, 70–77]
		IGH D-J usage: D2 (Most frequent: **D2-2**)	Characteristics of canonical SHM	GCB or non-GCB type DLBCL shows no association with clonal status of IG rearrangements	No correlation between DLBCL subtypes and relapse clonal evolution	
		Stereotyped HCDR3: **V**_**H**_**1-69**/D3-10/J_H_6 **V**_**H**_**1-69**/D3-3/J_H_6 **V**_**H**_**4-34**/D3-22/J_H_2	High IGL SHM with poorer prognosis	Shorter IGH-CDR3 is associated with better OS and PFS	Clonally-unrelated relapse tends to occur later after initial lymphoma	
			The degree of SHM in GCB is higher than in ABC	Ongoing IGH SHM correlates with poorer survival	Selective pressure including treatment selection before relapse and antigen selection during malignant transformation	
			SHM occurs in FR regions	Abnormal IgMκ/IgMλ ratio predicts worse prognosis		
			The overexpression of *BACH2* is associated with ongoing SHM of IGHV and more frequently happens in GCB subtype			
FL	B cells in GC	IGH V-D-J usage: V_H_ usage: V_H_3 > V_H_4 > V_H_1 (Most frequent: **V**_**H**_1**-18**, **V**_**H**_**3-48, V**_**H**_**3-15, V**_**H**_**3-34, V**_**H**_**3-23, V**_**H**_**3-30, V**_**H**_**3-21**) D usage: D2, D3 (Most frequent: **D3-10, D3-22, D3-3**) J_H_ usage: J_H_4	Ongoing SHM or highly mutated	Biased V_H_ usage indicates antigen participation in lymphomagenesis	ISFL: an intermediate stage between FL and t(14;18) B cells	[46, 49, 58, 59, 63, 71, 72, 75, 78–82]
		In tFL (compared with non-GCB DLBCL): V_H_1 is underrepresented and V_H_3 is overrepresented	t(14;18)(q32;q21), the BCL2/IGH rearrangement	The V_H_3-48 gene is associated with the risk of histological transformation (HT)	Transformation of FL to DLBCL more frequently occurs via divergent evolution from a putative common progenitor	
			Significant mutations in either HCDR3 or LCDR3 but not both	The N-gly sites within IGHV region	The transformation was achieved through HT and involved a clonal relationship between FL and the more aggressive disease	
				The natural course of FL is not linear	Patients with higher number of subclones have a longer PFS	
					BCR signalling is functional throughout FL tumour evolution	
**MM**	Memory B cells	1.IGH V-D-J usage V_H_ usage: V_H_3 > V_H_4 > V_H_1 (Most frequent: **V**_**H**_**3-30, V**_**H**_**3-23, V**_**H**_**5-51, V**_**H**_**1-69, V**_**H**_**3-9, V**_**H**_**4-31**; Absent: **V**_**H**_**4-34**) D usage: D3, D2 (Most frequent: **D3-3, D3-10**) J_H_ usage: J_H_4, J_H_6	Highly mutated CDR3 of either IGH or IGL with no intra-clonal variation	Higher SHM level is associated with an improved survival rate	Intra-clonal diversity of CDR3 sequences was rare	[15, 19, 44, 47, 81, 83–86]
		1.IGL V-J usage: V_κ_ usage: V_κ_1, V_κ_3, V_κ_2 (Most frequent: **V**_**κ**_**2-30, V**_**κ**_**1(D)-33**) J_κ_ usage: J_κ_4, J_κ_2 V_λ_ usage: No clear preference J_λ_ usage: J_λ_2, J_λ_3	Most cases are class-switched	CDR3 composition of MM disease clone resembled the normal immunoglobulin repertoire	All dominant clonal sequences were stable over time	
			Translocation involving IGH gene (14q32)	The success rate of IGK assay in λ-restricted samples is higher than in κ-restricted ones	Dominant clonal CDR3 sequences identified at baseline are reliable biomarker for MRD tracking	
			Less SHM in clonal V_κ_ rearrangement from λ-restricted clones compared with κ-restricted clones

### IG gene rearrangement pattern in ALL

ALL is considered to originate from pre-B cells, which are aberrantly blocked at the transition to immature B cells. This mechanism explains the unmutated or low-mutated status due to lack of SHM, the high frequency of unproductive IGH rearrangements due to continuously active recombinase enzyme, and the initiation of IGK/IGL rearrangements that go against allelic exclusion rules due to improper in-frame selection [40, 41, 47, 59]. Clonal evolution can’t be ignored in ALL and likely occurs by continuing rearrangement processes (successive VH to DJH or secondary rearrangements) [40, 41] and selection pressure mediated by treatments [60]. Measurements of the IG gene repertoire exhibited biased VH usage toward VH3 and VH1 families, most frequently involving the VH6-1, VH1-2, VH3-11, VH3-13, and VH3-15 segments. D2 and D3 families were overrepresented, in which the D2-21 segment was the most frequent. JH4 and JH6 were more frequently selected in JH families. In the Vκ family, Vκ1 and Vκ2 are preferentially used [40, 47]. Unfortunately, there is no obvious evidence indicating an association between IG gene characteristics and prognosis, but this conclusion is not yet validated due to the lack of large-scale studies.

### IG gene rearrangement pattern in MCL

The origins of conventional MCL (cMCL) cells and nonnodal MCL (nnMCL) cells are believed to be naïve mature B cells and memory-like B cells, respectively [44, 55]. The cMCL subtype generally exhibits an unmutated or minimally mutated CDR3 region [54]. The core mechanism in the malignant transformation of MCL has been demonstrated to be a translocation involving chromosomes 11 and 14 (t(11;14)(q13,32), *CCND1*/IGH), leading to the overexpression of CCND1 protein [5, 61, 62], while other cases may be driven by a *CCND2* or *CCND3* translocation with IGK or IGL [63]. Aberrant translocation events in MCL can occur in V(D)J recombination, SHM, or CSR based on the DSBs formed during these processes [44]. Similarly, a biased VH-D-JH repertoire has been observed, marked by the preferential use of the VH3, VH4, D3, D6, JH4, and JH6 families. In the VH repertoire, VH3-21, VH3-23, VH4-34, VH1-8, and VH4-59 were most frequently used, while in the D repertoire, D3-22 and D3-3 were overrepresented. The Vλ1, Vλ2, Vλ3, and Vκ3 families account for the highest proportion of the Vλ and Vκ repertoires, in which the Vλ2-14 and Vκ3-20 were the most frequent components. Stereotyped CDR3 regions of both heavy chain (HCDR3) and light chain (LCDR3) were addressed in MCL. The phenomenon of the narrowing of the VH repertoire and the occurrence quasi-identical receptors due to the involvement of a limited set of antigens in the development of lymphomas is referred to as “stereotyped” [51]. Several stereotyped forms of V(D)J combinations have been discovered and described, including VH4-34/D2-2/JH6, VH3-21/D3-9/JH6, VH3-21/D6-6/JH6 + Vλ3-19/Jλ2, Vλ3-19/Jλ2-1, Vλ2-14/Jλ2-1, and Vκ3-10/Jκ2-1 [51, 54, 55]. These features endow the MCL group with good suitability for IG-based clonality assessment.

### IG gene rearrangement pattern in CLL

Opinions on the origin of CLL cells are widely divided due to the existence of unmutated (U-CLL) and mutated (M-CLL) subtypes, classified by the level of SHM with a cutoff value of 2% [48, 49]. Currently accepted theories include transformation from immature B cells and B cells at an early stage of SHM, possibly resulting in U-CLL with more aggressive behavior, B cells exhibiting SHM in M-CLL with more benign features, or transformation from marginal zone B cells in both subtypes [64, 65]. SHM in CLL is concentrated in hotspots with limited intraclonal diversification, indicating the absence of an ongoing SHM process [52]. The VH1, VH3, and VH4 gene families were more preferentially used, in which the VH1-69, VH4-34, VH3-23, VH3-30, and VH1-2 segments were observed to be the most frequent [51]. The most striking characteristic of CLL is the high proportion of clones with stereotyped BCRs represented by VH1-69/D3-16/JH3 + VκA27, VH1-69/D3-3/JH6 + Vλ3-9, VH3-21/D3-3/JH6 + Vλ2-14, VH3-21 + Vλ3-21, VH4-39/D6-13/JH5 + Vκ(D)1–39, etc. BCRs involved with VH1-69 possess longer and unmutated CDR3, while BCRs involved in VH3-21 possess a shorter and less mutated CDR3 [48–52]. CLL cases with stereotyped and nonstereotyped BCRs may undergo different oncogenetic trajectories. Most major subsets of stereotyped BCR in CLL were unmutated with high conservation across the entire HCDR3. Moreover, studies have confirmed the coexistence of satellite subsets, defined by a small quantity and high similarity with consistent clinical profiles with major subsets [66]. Measurements of the IG gene repertoire of CLL have been demonstrated to have a certain value in diagnosis and prognosis prediction.

### IG gene rearrangement pattern in DLBCL

DLBCL, not otherwise specified (DLBCL, NOS), is classified into two distinct groups, the germinal center B-cell-like (GCB) subtype and non-GCB subtype, characterized by different cells-of-origin (COO) and responses to chemotherapies or targeted therapies [67]. Both subtypes of DLBCL display established features of canonical SHM, while GCB cases exhibit ongoing SHM associated with poorer survival, and non-GCB were considered mutated [48, 53, 56, 68]. High rates of SHM in DLBCL were verified in both the HCDR3 region and the LCDR3 region of BCR, with a worse prognosis in the former case and a better prognosis in the latter case [56]. SHM can also occur in the framework regions (FR), requiring complexity in the design of sequencing primers [69]. Moreover, Kikuchi et al. demonstrated that the overexpression of BACH2 was critical for ongoing SHM of HCDR3, and this phenomenon occurred more frequently in the GCB subtype, which further helped to decipher the molecular mechanism and its link to clinical behaviors [70]. Assessment of the IG gene repertoire showed more frequent use of VH1 and VH3, followed by VH4 and VH2. VH1-2, VH4-34, VH3-23, VH4-39, VH1-69, VH5-51, and VH3-21 segments were preferentially selected in DLBCL with a biased distribution in two COO subtypes, highlighted by the clustered highly mutated VH4-34 segments in the non-GCB subtype and VH3-21 segments more frequently used in unmutated cases [53, 69]. The D2, D3, JH4, and JH6 gene families were overrepresented, and D3-22 and D3-10 outnumbered the other segments. The D2 gene family and D2-2 segment were more frequently used in incomplete D-JH rearrangements. Evidence supports a stereotyped HCDR3 region in DLBCL, including VH1-69/D3-10/JH6, VH1-69/D3-3/JH6, and VH4-34/D3-22/JH2, summarized by Sebastián et al. [53, 56]. Clonally related relapses resulting from clonal evolution in DLBCL can be intricate, operating in either early-divergent mode or late-divergent mode, both proven clonally related as evidenced by the same V(D)J rearrangement. The early-divergent mode was named according to the behavior of the preexisting, chemoresistant subclones diverging early and developing in parallel with the major clone, characterized by more SHM sites in the relapse sample than in the diagnostic sample. The late-divergent mode is considered to occur at a later period of oncogenesis with subclones derived directly from the major clone that have fewer differences in SHM sites and number. However, there was no significant correlation between DLBCL subtypes and evolution-relapse mode. Several studies have also noted the selection pressure between remission and relapse, such as that resulting from treatments and antigens [68, 71–73]. Other IG repertoire-associated factors influencing the prognosis included monoclonality, the length of the HCDR3, and the abnormal ratio of functional IGK/IGL rearrangements [56, 69, 74, 75].

### IG gene rearrangement pattern in FL

FL cells originate from GC B cells, marked by t(14;18) (q32;q21) (IGH/BCL2), resulting in the overexpression of BCL2 [76]. Recently, several studies have stressed the concept of in situ FL, an intermediate stage between FL and normal t(14;18) B cells, as an origin of FL [45]. The acquisition of asparagine-X-serine/threonine (N-gly) sites in the IGHV region has been recognized as one of the early initiating events of FL pathogenesis and a stable, conserved, and essential hallmark for the survival, proliferation, and dissemination of FL cells [45, 77]. The CDR3 of either IGH or IGL in FL cells is highly mutated, with significant intraclonal diversity caused by continuous exposure to GC [45, 57, 76, 78, 79]. Similar to the other B-lineage malignancies mentioned above, the VH3, VH4, and VH1 gene families were more frequently used in FL, in which VH1-18, VH3-48, VH3-15, VH3-34, VH3-23, VH3-30, and VH3-21 were preferentially selected. D3-10, D3-22, and D3-3 accounted for most of the D2 and D3 gene families used in FL. JH4 was the most frequent JH component [58, 69, 80]. Interestingly, FL can transform into other more aggressive malignancies, usually DLBCL. The transformed FL exhibits a clonal relationship with the original FL and changes through histological transformation, involving continuous BCR signaling, possibly associated with the overrepresented VH3 gene family, especially the VH3-48 segment [80]. Additionally, FL-transformed DLBCL tends to occur through a divergent pattern from the common progenitor rather than via direct linear evolution [73].

### IG gene rearrangement pattern in MM

Compared with other B-lineage lymphomas, MM possesses more stable properties at the molecular level due to its origin from plasma cells, which have completed the GC reaction and consequently exhibit more mature features. CDR3 regions of either heavy chain or light chain in MM cells are highly mutated without intraclonal variation, while most MM secretes class-switched Igs, indicating the initiation of pathogenesis at the relatively late phase of the GC reaction [46, 79, 81, 82]. A higher level of SHM was associated with better OS outcomes [18]. The ontogeny of MM was also demonstrated to possibly result from translocation events involving the IGH gene (14q32) caused by aberrant V(D)J recombination, CSR, or SHM [43]. The IG repertoire, the relationship between Ig secretion, and the corresponding V(D)J recombination patterns in MM resemble normal cells [18, 83]. The VH3, VH4, and VH1 gene families are more frequently used, and VH3-30, VH3-23, VH3-9, VH4-31, VH1-69, and VH5-51 are the most frequent. Interestingly, the autoreactivity-associated component, the VH4-34 segment, is completely excluded from the IG repertoire of MM, indicating an intrinsic anti-autoimmunity quality. The D3-10 segment in the overrepresented D2 and D3 families occurs most frequently. JH4 and JH6 are again preferentially used in MM [79, 83]. In the IGK/IGL repertoire, Vκ1, Vκ2, Vκ3, Jκ4, Jκ2, Jλ2, and Jλ3 are more frequently chosen, while no clear preference for Vλ segments was observed. Vκ2-30 and Vκ1(D)-33 account for a sizeable portion of the Vκ repertoire [46, 83]. More IGK rearrangements, including Kde-mediated deletions, and less SHM in the IGKV regions, were demonstrated in λ-restricted cases than in κ-restricted cases, consistent with normal allelic exclusion [14, 84]. Clonal evolution in MM is rare, indicated by the stability of dominant sequences identified at diagnosis over time [18]. MRD monitoring of MM through IGH-based clonality assessment is feasible due to disease progression without variation at the molecular level, and the sensitivity could be further improved by the addition of IGK panels [83].

## MRD monitoring through clonality assessment by NGS

Because almost all B-lineage malignancies have distinct and stable V(D)J recombination patterns, the BIOMED-2 protocol was first designed by a European BIOMED-2 collaborative study as a PCR-based technology for routine clonality diagnostics [85]. This classical method relies on multiplex PCR with 97 standardized primers designed for amplification of different Ig/TCR gene segments, and the PCR products can be analyzed for clonality assessment by heteroduplex analysis or GeneScanning [29]. High-throughput sequencing techniques have been rapidly developed and upgraded. The Lymphotrack assay was established by combining the basic strategy of BIOMED-2 and NGS technology. After input, the compatible FASTQ files can be processed into fully analyzed data by the corresponding application Lymphotrack DataAnalysis [86]. Productive rearrangements were further analyzed for parse, re-organization, and exportation using algorithms. In this process, a clonotype was established in the tumor sample using locus-specific primer sets for IGH-V, -D, and -J rearrangements and the Miseq Illumina platform. The output form of results was then further analyzed based on the international ImMunoGeneTics (IMGT) information system to identify the exact V–D–J sequence and the corresponding frequency. Information was ultimately reported as Ig gene repertoires, VH CDR3 length, exact amino acid sequence and frequency of SHM. Generally, a clonotype with a frequency of higher than 5% of all rearranged V(D)J sequences were identified as a malignant clone. The malignant clone with the highest frequency in the baseline sample was named the “index” clone, and was tracked in the follow-up samples for the MRD measurement. Standard and automated data processing can be performed easily, locally and securely, making it feasible in most laboratories. In addition, a similar product, ClonoSeq from Adaptive Biotechnologies [87], has already been approved by the FDA for MRD tracking in ALL and MM.

Considering the abundance of information produced by the NGS IG method, it is plausible and practical to apply this strategy for clonality assessment in diagnosis at baseline and MRD monitoring follow-up assessments.

### Importance of MRD monitoring in clinical practice

The variance of clinical remission (CR) among different regimens and diseases has revealed the limitation of the current definition by clinical manifestation and imaging tests, raising a claim for a more stringent version. MRD is generally acknowledged as one of the most powerful approaches for the prediction of relapse and prognosis. MRD-positive patients have far less favorable event-free survival outcomes than MRD-negative patients [10–12, 15, 22, 88–91]. The prognostic value of MRD among patients undergoing specific treatments is mainly reflected in the dynamic risk-stratification ability. Moreover, by serially monitoring the clearance of tumor cells in the BM or PB during and after chemotherapy or novel immunotherapies, the modulation of treatment duration and intensity can be prompted and executed by either early termination/intensification of treatment in patients who remain MRD-positive or after interruption of continuous treatment in patients who become MRD-negative. Measurement of MRD at the end of therapy also assisted in identifying cured or optimally treated patients, in turn providing an evaluation of specific therapeutic effects [61, 92]. Methods for the effective application and integration of MRD monitoring in clinical practice for relapse prediction were established on the essential premise that (1) molecular relapse precedes clinical relapse by a time interval long enough for intervention and (2) instant intervention initiated during the lead time influences the outcome and results in a better prognosis [61].

However, MRD monitoring is not widely available in lymphoma care despite the benefits shown in therapeutic outcomes and scientific research. Several obstacles have impeded the improvement of MRD and the ability to obtain feedback from real experiences, including the lack of incorporation of MRD monitoring in prospective trials involving novel treatment, the relatively high cost of high-throughput sequencing per sample, the long delays between technical evolution and the ultimate outcome (e.g., OS), the labor and special care required in the trial and the complex statistics required for analysis [61]. Furthermore, the conclusion inferred from MRD monitoring can be obscure due to the nonuniform standard of MRD negativity across laboratories. MRD negativity is typically defined as the absence of clonal malignant cells in BM aspirates with a minimum sensitivity of 1-cell in ≥ 10^5^ nucleated cells [10, 22]. However, recent studies provided evidence for more OS and PFS benefits and better relapse prediction capabilities when a threshold of 1 × 10^–6^ is used [14, 93, 94]. A more sensitive, repeatable, and multifunctional method for MRD monitoring is urgently needed.

### The process of IG NGS-based clonality assessment in MRD monitoring

**Fig. 3:**
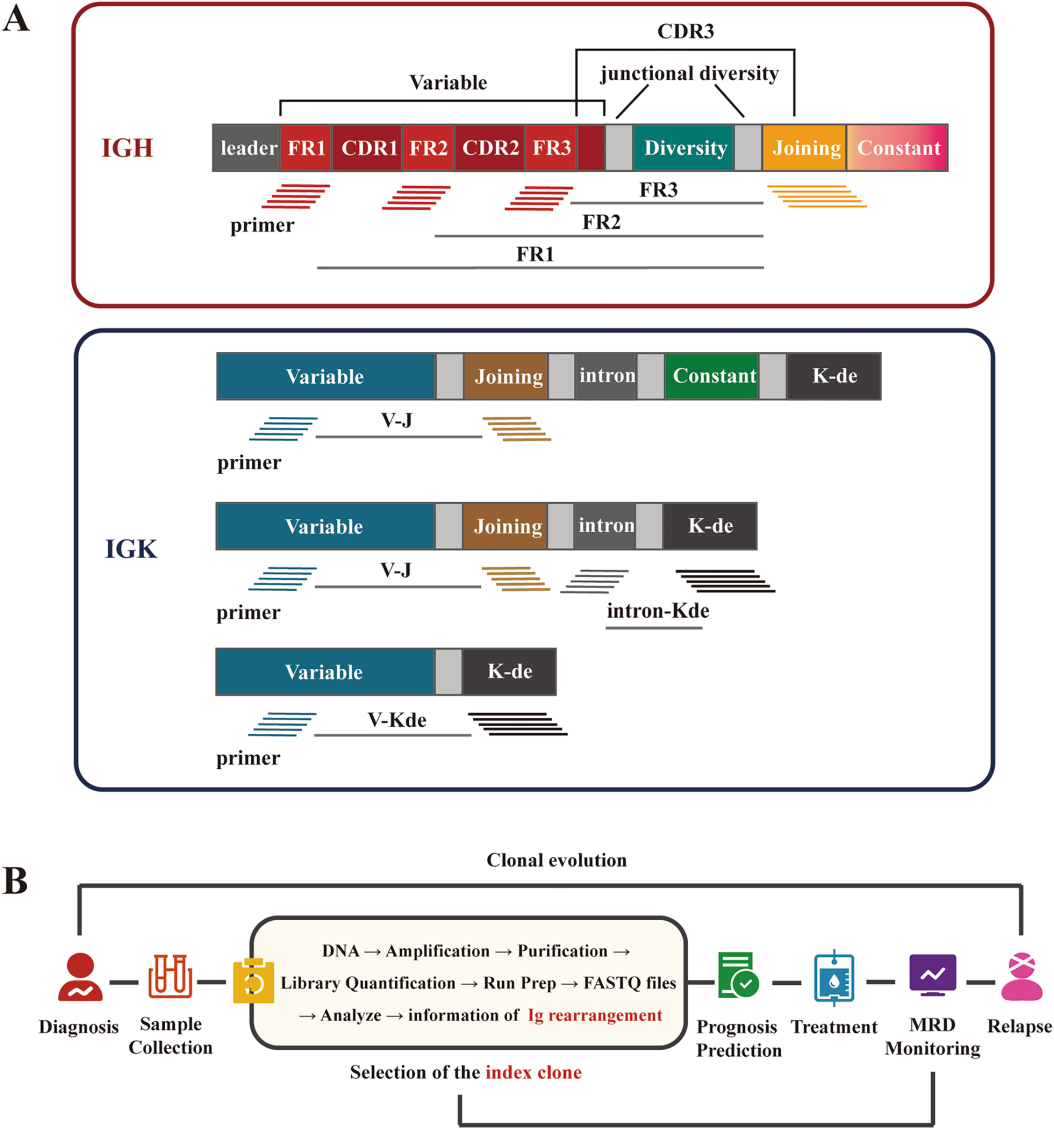
Strategy of the IG-based NGS method. **A** Pairs of primers for IGH and IGK sequencing. The forward primers target FR1, FR2 and FR3, while the inverse primer targets the JH region in IGH. 3 pairs of primers were designed for IGK sequencing, including the forward primer targeting Vκ or introns and the inverse primers targeting Jκ or Kde. **B** The workflow of NGS MRD monitoring. After diagnosis by the gold-standard, the samples of patients are collected (BM or PB) and sequenced to identify index clones. Information acquired during sequencing can also be used in risk stratification and prediction of prognosis. By tracking the index clone, the MRD level is measured continuously during and after the treatment. The major clones in samples from patients who experience relapse are compared with the index clone at diagnosis to study clonal evolution

The complete process of IG-based diagnosis and MRD monitoring by NGS in lymphoma is shown in Fig. 3 B. After the initial diagnosis of B-lineage malignancy was confirmed by clinical symptoms, imaging manifestations, and histopathological examinations, the BM aspirate samples were preserved and subjected to high-throughput sequencing. Index clones were identified in these samples by the following criteria: (1) the proportion of index clones needs to be at least 3% of all sequences at the specific locus, (2) the frequency of cells that carry index clones needs to be at least 0.2% of all nucleated cells [5], and (3) other criteria in kits designed by different companies [14]. By using the algorithm of exact match and up to 2-bp mismatches, the disease clones in follow-up samples were compared with the initial index clones in diagnostic samples, based on which the presence or absence of MRD was identified, and the quantity of MRD was calculated [18]. By analyzing the IG repertoire in the sequenced sample, including V–J usage and SHM levels, the disease of a specific patient can be further classified at a molecular level and ranked in order of the degree of risk. The index clone was tracked in a series of BM aspirations during and after treatment for the dynamic evaluation of therapeutic effects and prognostication, including relapse prediction. When tracking multiple clonotype sequences, it is important to consider the type of gene rearrangement being tracked. A check of the tracking sequence in a negative control is also necessary to ensure that it is not a part of the polyclonal background, which could lead to a false-positive result. It should also be noted that the adequacy and tumor infiltration level of the specimen required for the identification of an index clone at baseline is relatively high for sufficient DNA input to reach a sensitivity of at least 1 × 10^–5^. The functionality of MRD monitoring based on this assay can be influenced by several technical limitations of sampling and sequencing, including the amount of input DNA, the cellularity of a BM sample, and possible significant overestimation of residual tumor cells due to the calculations (detectable index clonal sequences/total IGH/IGK sequencing reads) [5, 18].

The strategy designed to amplify specific regions in the IGH/IGK gene is based on a set of consensus primers targeted at the conserved FR region (Fig. 3A). For IGH sequencing, the forward primers target FR 1 to 3 (FR1, FR2, and FR3) in the VH region, while several consensus reverse primers target the JH region. For IGK sequencing, primers target the VK-JK, VK-Kde, and INTR-Kde gene rearrangements. IGL sequencing is rarely applied [14, 19]. Targeted sequences are amplified and purified, based on which the DNA library is established and optimized by intense quality control. The output of sequencing and alignment, usually formatted as a FASTQ file, is analyzed and sorted to identify index clones by predetermined criteria.

For some cases, the IGHV mutational status plays an important role in prognosis prediction, such as in CLL. The matched bioinformatics software Lymphotrack IGH FR1 Assay Master Mixes can be used to meet the needs for evaluating the SHM rate of the IGHV region. The IGHV mutational status will be determined based on the percent of mismatch between the clonal amplicons and the corresponding germline reference genes, the prediction of in-frame or out-of-frame translation, the possibility of a premature codon introduced by the mutation, and the percentage of VH gene coverage in the region targeted. SHM, in turn, may hamper the identification of clonal immunoglobulin rearrangements due to the possible prevention of primer annealing during multiplex PCR. Nonetheless, to identify distinct IGHV genes in the IGHV subgroup, commercial kit software will require a match using the IgBLAST or IMGT database and list the candidate IGHV genes in order of identity. The best match is preferentially chosen for subsequent analyses. The result will be considered dubious and excluded when a stop codon or frameshift mutation is produced. Although the bias resulting from SHM is hard to avoid, the comparison step ensures the validity of the interpretation as much as possible.

The information presented in the analysis report is as follows: (1) a summary of a sorted list of unique sequences, including the best-matched V–J family recombination event, length of the read, the actual and cumulative rate of the unique read in total reads, coverage of the identified V-gene by the unique read, the mutation rate to partial V gene and the prediction of in-frame mutation and stop codon; (2) graphs of V–J usage/sequence frequency and (3) a FASTA file of the unique sequences sorted by count.

### Comparison of the performance of techniques applied in clonality assessments and MRD monitoring

The advantages and drawbacks of the methods for clonality assessment are presented in Table 2 in detail [5, 29, 60, 72, 78, 93, 95–100].

**Table 2:** Comparison among techniques used in clonality assessment by IG rearrangements

Techniques	ASO-RQ-PCR	BIOMED-2 Strategy	NGS
Samples	DNA, including low-quality DNA from small biopsies and FFPE tissues (low amplification efficacy)		DNA, high-quality DNA is required at diagnosis
Mechanism	Consensus primers are used to sequence and design precise primers for specific amplification of rearranged fragments	Multiplex PCR and capillary electrophoresis (GeneScan), 97 new primers	Obtain all information of the IG rearrangements then compare the results with germline sequences
Sensitivity	1 × 10^–4^ ~ 10^–5^	1 × 10^–3^	1 × 10^–6^
Clonal evolution	Cannot be detected	Present or absent	Sequences can be detected
Standardisation	Poor-standardised	Well-standardised	Well-standardised
Specific request	Design patient-specific primers by sequencing the junction region of first PCR products	Analysis of PCR products: monoclonal (1–2 peak) polyclonal (Gaussian distribution)	Higher DNA input is needed for higher sensitivity
Advantages	Relatively high sensitivity	Wider application range	Higher sensitivity and resolving power
	Fewer technical requirements	Good reproducibility	More convenient and rapid operation: synchronized detection, serial monitoring during follow-up
	Short turn-around time	High accuracy	Relatively objective interpretation
	Economical and affordable	Low DNA requirements	EuroClonality-NGS working group
	Suitable for MRD monitoring	Already commercialised and instituted in most laboratories	Better identification of bi-allelic rearrangements and oligoclonality
	Multiple target genes increase the accuracy	Recommended as standard method for clonality assessment in lymphoproliferative diseases	Bioinformatic identification and correction
	Established guidelines for the analysis of RQ-PCR data		Monitor MRD status using peripheral blood
Disadvantages	Pseudo-clonality (false-positive) and oligoclonality (weak clonal products) due to non-specific amplification and insufficient discernibility		On-going optimisation of techniques
	Mismatches (false-negative) due to SHM		Criterions for statistics analysis are not consistent
	Time-consuming and labour-intensive	Unsuitable for MRD monitoring	Applied in limited laboratories
	High standards of experimental condition	Separate PCR products by the lengths but not the sequences	High requirements for DNA input quality at diagnosis
	Lack of sufficient diagnostic materials which may influencing the standard curve	Separate PCR products by the lengths but not the sequences	Well-functioning networks and collaboration between centres are needed
	Not suitable when clonal evolution or a secondary malignancy occurs, or tumours originate from immature cells	Cumbersome operations due to the multi-step approach	A large-scale validation study is needed
		Do not harbour correction mechanisms	

The gold standard for clonality assessment is the BIOMED-2 multiplex PCR-based protocol for IG/TCR targets, but it is not suitable for MRD monitoring [101]. The workflow, experimental conditions, and analysis methods were standardized in the BIOMED-2 protocols. Monoclonality, which represents malignant proliferation, is displayed as 1–2 peaks in the background, while polyclonality, which indicates physiological immune reactions, has a Gaussian distribution.

ASO-RQ-PCR can be applied for diagnosis and MRD monitoring of lymphoma with a sensitivity of 10^–4^ ~ 10^–5^. In this approach, IG regions are targeted and amplified by consensus primers and then sequenced to design more precise, patient-specific primers and probes [95]. ASO-RQ-PCR provides quantitative results recorded by fluorescence signals, making it suitable for MRD measurement. Given that the design of patient-specific primers and probes is complex, labor-intensive, and cannot be standardized, ASO-RQ-PCR is difficult to apply widely in multiple centers.

Compared with the BIOMED-2 protocol and ASO-RQ-PCR method, the IG-based NGS approach performed better on many levels, such as higher sensitivity, the ability to obtain more information about the IG repertoire, and the ability to track clonal evolution; additionally, the workflow is well-standardized (Table 2). Since the sensitivity of the BIOMED-2 method was 0.1%, which partially hampered its utilization for MRD tracking, the comparison between BIOMED-2 and NGS mostly focused on the concordance of the types of clonality or the sequences detected. The EuroClonality-NGS working group tested the suitability of NGS-based IG rearrangement detection in frozen and formalin-fixed paraffin-embedded specimens (low-quality DNA) using the ARResT/Interrogate platform for data analysis. Accurate clonotypes in all healthy samples were successfully identified by NGS, while dominant IG gene rearrangements identified by NGS were identical to those identified by BIOMED-2. The NGS-based method also identified 22% more clonal rearrangements that were not detected by the conventional method, possibly due to the new design of shorter amplicons [101]. A multicenter study compared these two technologies based on 209 specimens of reactive and malignant lymphoproliferation and validated the high interlaboratory concordance (99%) of NGS-IG detection and its high concordance (98%) with the BIOMED-2 method for the exact sequences and clonality. An even higher sensitivity was obtained with the NGS-IG method than the gold standard, inferred from a higher detection rate in diagnosed lymphoma samples. Satisfactory resolution of reactive and malignant samples was also achieved by NGS-based clonality tests [102]. Similarly, several other studies established for testing the NGS-based IG clonality method in routine clinical practice unveiled superior performance of NGS, marked by a clonality detection rate of 97% and high concordance (96%) with capillary electrophoresis assays at diagnosis [103] and a much higher positive rate obtained by NGS compared with fragment analysis in follow-up samples for MRD detection [84]. Less efficient identification of clonal IG rearrangements by NGS in HL compared with NHL was observed in a study with a small sample size; however, the NGS IG method performed better than BIOMED-2 [104]. Overall, the NGS-based method is superior to BIOMED-2 for clonality detection.

FC-based methods, including MFC and NGF, are considered the gold standard of MRD measurement in clinical practice with a wide applicable range, short turnaround time, reliable results, and relatively low cost. Malignant cells in samples are identified and quantified based on aberrant immunophenotypes labeled with different colored antibody signals. The sensitivity reaches 1 × 10^–4^ in MFC and 2 × 10^–6^ in NGF [15, 20, 21]. However, FC-based approaches are limited by technical defects and expertise requirements [105]. First, the analysis of FC results requires a high level of expertise to avoid possible subjective interpretation, while variations in instrument settings and reagents (antibodies and fluorescein) are unavoidable. Second, FC is complicated by the change or loss of the surface markers selected during monitoring resulting from clonal evolution and targeted therapies such as CAR-T cells. Low tumor burdens, sample hypocellularity, and diseases lacking specific markers, such as DLBCL, also limit the application of FC. The relatively low sensitivity of MFC limits MRD detection after intensive treatments (false-negative results), while false-positive results may arise after the induction of immune reconstruction. Third, the fast turnaround time was paralleled with the requirement of fresh instead of cryopreserved samples, which are not available for retrospective studies. Last, FC results lack stringent quality control. These limitations were partially overcome by the advent of NGF, in which the sensitivity was significantly improved, the workflow was standardized following the EuroFlow guidelines, and the results were controlled based on the detection of hemodilution [17, 88].

Another PCR-based method, the droplet digital PCR (ddPCR) assay, can also be applied for MRD monitoring. ddPCR was proven to be an efficient method for MRD monitoring in malignancies with distinct translocation/fusion transcripts/recurrently mutated gene markers, such as BCL/IGH rearrangements in FL [106], BCR-ABL fusion transcript in chronic myeloid leukemia [107, 108], MYD88 L265 mutation in Waldenstrom macroglobulinemia[109] and NPM1 mutation [110] or IDH1/2 mutation [111] in AML. ddPCR solves some of the disadvantages of quantitative PCR, including establishing a standard curve and positive cases with unquantifiable results while maintaining a sensitivity of 1 × 10^–5^. Furthermore, several studies demonstrated that ddPCR had a good concordance with RT–qPCR, but its ability to quantify the level of markers was more precise [108, 112]. Based on water–oil emulsion technology, ddPCR fractionates the sample into nanoliter-sized 20,000 droplets and amplifies the template molecules in each droplet [113]. The high partitioning endows ddPCR with highly sensitive and reliable absolute quantification capability [107, 114]. However, as an approach developed to quantify specific leukemic aberrations, ddPCR is still strictly dependent on qualitative nested PCR as the marker screening tool. The prevalence of the specific hallmarks (absent in 35–40% of patients) significantly limits the utilization of ddPCR in most hematological diseases, making this PCR-based method suitable for only a minority of patients [114, 115]. High mutation rates and translocations or mutations unrelated to tumors can also influence the final interpretation of results. The ddPCR method can be time-saving, cheaper, and easier to perform when compared with NGS-based methods, but it only detects genetic alterations that have already been identified, while NGS provides more comprehensive information [116]. Additionally, the primers used for ddPCR need to be specifically designed for patients based on sequencing results.

The comparison of FC-based, NGS-based, and PCR-based approaches is shown in Table 3 [18, 20, 100, 106, 109, 115, 117–122]. The unique advantages of NGS-based techniques stand out from the other approaches to MRD monitoring. The NGS method exhibited superior detection for cases with lower tumor burdens, minor subclones, and a high level of SHM at diagnosis. MRD can be better defined by tracking the behavior of specific clones through NGS, providing a more accurate prediction of relapse and more evidence of clonal evolution. Moreover, biclonalities, oligo-clonalities, and uncommon rearrangements can also be identified with reasonable confidence. The IG NGS approach provided a common picture of not only the MRD of malignancies but also the immune repertoire. Similar to PCR-based approaches, the IG NGS method can be used in cryopreserved and fixed samples. The objective interpretation and the automated analysis of subsequent samples based on pipelines and predefined thresholds further expanded the scope of its application. Limitations of the IG NGS approach exist, such as the higher standard for accessibility caused by requirements for better computing and high-quality DNA input at diagnosis, longer turnaround time of approximately 5–7 days, failures of detection due to the indistinguishable background of reactive B cells, V deletions and incomplete DJ rearrangements or hemodilution, and the lack of well-established, widely accepted protocols. NGS technology is based on an initial PCR step, which could be influenced by annealing and SHM in primer-binding regions. The high cost per sample is also a constraint, preventing the use of NGS-based MRD monitoring in most clinical trials.

**Table 3:** Comparison between flow cytometry and IGH/IGK rearrangements identified by NGS in MRD monitoring

Items	Multiparameter Flow Cytometry	IG NGS-based Clonality Assessment	Droplet Digital PCR
Information offered	Proportion of cells, morphological features, immunophenotypic characteristics	Genetic alterations, immune repertoire	Genetic alteration, breakpoints involved in specific translocations
Turn-around time	24–48 h	5–7 days	24–48 h
Sample type	Bone marrow aspirates (more frequently) or peripheral blood	Bone marrow aspirates or peripheral blood	Bone marrow aspirates or peripheral blood
Sample quality	Fresh samples acquired within 24–28 h or DMSO-preserved samples	Fresh samples or preserved samples (FFPE, cryo-preserved samples, etc.)	Fresh samples or preserved samples (FFPE, cryo-preserved samples etc.)
Sample quantity	Relatively large (1 × 10^5^ ~ 1.5 × 10^6^ mononuclear cells) [118]	Small, but high DNA input is required for the identification of index clones (DNA input of 40–200 ng) [117]	Small, suitable for cases with low tumor burden or positive but not quantifiable qPCR results (DNA input of at least 150 ng) [119]
Application range	≥ 95% of patients [111, 119, 120]	Approximately 100% of patients [19, 24, 117]	minority of patients, dependent on the target selected [112, 113]
Sensitivity	1 × 10^–4^(MFC), 2 × 10^–6^(NGF) [16, 18]	1 × 10^–6^ [19]	1 × 10^–5^ [110]
Operation procedure	Simplified steps	Relatively complicated steps	Relatively complicated steps
Analysis and Interpretation	Subjective, a high level of expertise is required	Objective, the analysis is automatically completed by the software	Objective, the analysis is automatically completed by the software
Clonality assessment	Clonal heterogeneity at the genetic level cannot be detected, but cell heterogeneity can be identified	Subclones and clonal evolution at the genetic level can be identified	Clonal heterogeneity at the genetic level and cellular level cannot be detected
Cost	Relatively cheap	Expensive	Relatively expensive

### The superiority of NGS-MRD in clinical practice

Statistics from clinical trials in which treatment decisions were made based on MRD are scarce due to the obstacles to practically applying MRD monitoring. However, several studies have focused on the functionality of the NGS-based MRD method by comparing it with other gold-standard approaches in registered clinical trials evaluating different B-lineage malignancies (Table 4). In summary, NGS-based MRD exhibited excellent performance in sensitivity, precision, reproductivity, and prediction of relapse. This method showed at least a comparable ability to identify the accurate sequence compared with Sanger sequencing [18, 98, 123] and could define and track the index clone compared with ASO-PCR [11, 22, 93, 124], MFC [15, 125], NGF [18, 20, 21, 123] and mass spectrometry [118]. With further optimization for satisfactory quantification and higher economical efficiency in the future, NGS-based MRD monitoring has the potential for wider application in routine clinical practice.

**Table 4:** Performance of MRD monitoring by IGH/K rearrangement in different B cell malignancies

Authors	Disease (Sample size)	Samples (Sample size)	Treatment/Clinical trial	IG rearrangement detection for index clone			MRD detection in follow-up samples			Conclusion
				Cases detected by IG NGS	Cases detected by other techniques	Concordance in detected sequences	MRD status detected by IG NGS	MRD status detected by other techniques	Concordance in MRD status detected by both techniques	
Genuardi et al. [96]	MCL(20)	BM(10) or PB(10)	Phase III MCL0208	95% (19/20)	Sanger sequencing: 75% (15/20)	87% (13/15)	Not available	Not available	Not available	NGS-based IGH screening might have the ability to track major clones in MRD monitoring
Ladetto et al. [91]	ALL(15), MCL(30), MM(10)	BM(218) or PB(160)	Prospective clinical trials	ALL: 100%(15/15) MCL: 86%(26/30) MM: 80%(8/10)	ASO-PCR: ALL:100%(15/15) MCL: 73%(22/30) MM: 80%(8/10)	95.5%(41/43)	Not available	ASO-PCR, not available	Fully concordant: 79.6% (211/265) Discordant: 20.4%(54/265), with 1.5% (4/265) major qualitative discordance, 5.3%(36/265) borderline qualitative discordance and 5.3%(14/265) quantitative discordance	NGS used in the identification of IGH clonotypes provides results that are at least comparable to ASO-PCR
Pulsipher et al. [122]	ALL(56)	BM (41 for pre-HCT analysis, 125 for post-HCT MRD)	Trial ASCT0431	100% (41/41)	Not available	Not available	Relapse probability is 0% (0/22) and 53% (9/19) for pre-HCT NGS-MRD- and pre-HCT NGS-MRD + patients, respectively Relapse probability is 25% and 67% for post-HCT NGS-MRD- and post-HCT NGS-MRD + , respectively	FC: Relapse probability is 16% and 46% for pre-HCT MFC-MRD- and pre-HCT MFC-MRD + patients	11 patients with post-HCT NGS-MRD + and post-HCT MFC-MRD- relapsed; none of patients with post-HCT NGS-MRD- and post-HCT MFC-MRD + relapsed	IGH V(D)J NGS-MRD predicted relapse and survival more accurately than FC-MRD
Ho et al. [19]	MM(251)	BM(438)	Treated at MSKCC	93.6% (235/251)	EC and Sanger sequencing: 93.6%	100%	78.6% (147/187) of the MRD samples with an IG NGS-MRD + status	81.8% (153/187) of the MRD samples with an hsFC-MRD + status	concordance of 92.9% (170/183) in MRD status detected by NGS and hsFC	NGS and hsFC performed similarly, showing a high concordance rate
Medina et al. [21]	MM(106)	BM()	Spanish GEM2012 clinical trial	Not available	Not available	Not available	50% (53/106) of patients with an IG NGS-MRD- status	54.7% (58/106) of patients with an NGS-MRD- status	Good correlation between the two methods (r = 0.951, R^2^ = 0.905) with 15 discordant cases (5NGF + /NGS-; 10 NGF-/NGS +)	NGS has the excellent applicability and comparable results to NGF
Avet-Loiseau et al. [22]	MM(1085)	BM	Phase 3 CASSIOPEIA study	Not available	Not available	Not available	344 patients achieved an IG NGS-MRD- status	582 patients achieved a MFC-MRD- status	Good overall agreement was achieved in 83.5% of 733 patients evaluated by both NGS and MFC	NGS and NGF perform similarly in evaluating MRD regardless of response and CR status
Li et al. [121]	ALL(258)	BM or PB (258)	Ma-Spore ALL 2003 and ALL 2010 studies	497 disease clones in 90.3% (233/258) patients	Sanger Sequencing: 348 disease clones in patients	90.8% of clones detected by Sanger sequencing were identified by IG NGS	78% (54/69) of samples with quantifiable MRD detected by IG NGS	58% (40/69) of samples with quantifiable MRD detected by RQ-PCR	40/69 of samples with quantifiable MRD detected by both IG NGS and RQ-PCR, 15/69 of samples with negative MRD detected by both methods	Sub-clonal disease can be uncovered by IGH NGS compared with Sanger sequencing; IGH NGS shows improved sensitivity compared with RQ-PCR
Kriegsmann et al. [16]	MM(125)	BM(125 pairs)	Multi-centre prospective phase III HD6 trial	Not available	Not available	Not available	74.4% (93/125) of patients had an IG NGS- MRD + status	48% (60/125) of patients had a FC-MRD + status	68% (85/125) cases exhibited concordant MRD status detected by IG NGS and MFC	There exists good concordance between NGS and FC at a threshold of 10^–5^
Langerhorst et al. [116]	MM(41)	BM(NGS, 81 Or PB(MS, 82)	IFM-2009 clinical trial	Not available	Not available	Not available	18.5%(15/81) of samples were IG NGS-MRD-	21% (17/82) of samples were MS-MRD-	79% (64/81) of paired samples showed concordant MRD status detected by IG NGS and MS	MS is at least as sensitive to detect MRD compared with NGS and is alternative to NGS-MRD
Takamatsu et al. [12]	MM(125)	BM(125)	High-dose melphalan plus ASCT	An overall clone identification rate of 90% (113/125) by IG NGS method	An overall clone identification rate of 66% (75/113) by ASO-PCR method	Not available	Not available	ASO-PCR, not available	35 samples are NGS-MRD + /ASO-PCR-MRD- status; Patients with IG NGS-MRD + /ASO PCR-MRD- status (11) showed worse PFS than patients with IG NGS-MRD- status (7)	Low level MRD detected by NGS but not ASO-PCR has significant prognostic value
Yao et al. [23]	MM(4)	BM(11)	VTD/PAD induction + ASCT + thalidomide maintenance	Disease clones were detected by IG NGS in 100% (4/4) of diagnostic samples	Disease clones were detected by ASO-PCR and Sanger sequencing in 100% (4/4) of diagnostic samples	Disease clones detected by the two methods were 100% same	5 samples achieved MRD + status and 2 samples achieved MRD- status by IG NGS	5 samples achieved MRD + status and 2 samples achieved MRD- status by ASO-PCR	100% of the 7 follow-up samples achieved a concordant MRD status detected by IG NGS and ASO-PCR method	NGS yields MRD measurements concordant and comparable to ASO-PCR; NGS shows improved sensitivity
Medina et al. [24]	MM(101)	BM	GEM2012 MENOS65 clinical trail	Clonality was confirmed in 100% (101/101) of cases with IG NGS	Clonality was confirmed in 99% (100/101) of cases with Sanger sequencing	97.9% (93/95) of the disease clones detected by IG NGS and Sanger sequencing were concordant	Not available	NGF, not available	High correlation (R^2^ > 0.8) was maintained between NGF and NGS performed in each center, Only 14% (13/93) of cases were discordant: 4 NGS-MRD- and NGF-MRD + cases, 9 NGS-MRD + and NGF-MRD- cases	NGS is a suitable strategy for clonality and MRD detection with results comparable to gold standards (NGF and Sanger sequencing)

## Current challenges in IG NGS-based MRD monitoring

MRD monitoring has already become a relatively mature and widely acceptable technology despite insufficient reliability when independently guiding treatment-associated decisions. However, the answers to several questions remain obscure or controversial. The first is the significance of sensitivity in real-world utilization. Theoretically, the deeper the detection is, the more accurate the results will be, which was emphasized in some articles that demonstrated a higher relapse rate of NGS or NGF MRD-positive patients compared with those assessed with other techniques with lower sensitivity [11, 125]. However, those MRD-positive patients with stable and nearly disease-free status with no evidence of relapse, common in clinical trials evaluating novel treatments, were ignored in most studies, raising the question of whether the presence of MRD detected by the instruments themselves or the quantity of MRD above a predefined threshold influences the prognosis. Another factor that should be considered is the sample requirement. A substantial concentration of DNA input is needed to reach a sensitivity of 10^–6^, which is usually unavailable from patients who have experienced intensive treatment elsewhere and is also time-and labor-consuming. Although it is difficult to find and achieve the right balance of economic benefits and optimal results, the combination of higher sensitivity for detection and risk stratification based on the number of residual tumor cells can be an inspiration to develop next-generation techniques for MRD monitoring. The second question is associated with the sample types. Diagnosis and MRD monitoring are generally performed by analyzing the infiltration of tumor cells in the BM. It should be noted that BM aspiration and biopsy are invasive procedures with potential risks, limiting the ability of doctors to take repeated samples.

## Future prospects of IG NGS-based MRD monitoring

Compared with those for examining BM aspirations, tests for peripheral blood are more convenient and accessible. Recently, liquid biopsy technology has emerged to capture information about SHM, V(D)J rearrangements, amplification and gene copy variations. By using circulating tumor cells (CTCs), circulating tumor DNA (ctDNA), cell-free DNA (cfDNA), or other cell-free nucleic acids (mRNA, microRNA), liquid biopsy can be conducted in a noninvasive manner [126]. The results of several studies have suggested that ctDNA and cfDNA alone are practical for most lymphomas [5]. The rapid clearance of cfDNA allows tracking of the dynamic changes in MRD [61]. Unfortunately, the cfDNA level was proven insufficient as an independent prognostic factor in some studies [127]. The IG NGS-based method can also be applied to detect ctDNA and peripheral blood mononuclear cells, which is particularly promising for application in DLBCL [57, 128]. However, this approach is limited by the very low concentration of ctDNA or CTCs early in the disease, more localized infiltration of tumors, and MRD monitoring after intensive treatment; thus, liquid technologies inevitably present considerable and ongoing challenges requiring the development of ultrasensitive techniques [129].

## Conclusion

This review provides a comprehensive evaluation of IG NGS-based MRD monitoring, including the necessity of MRD monitoring, the scope of application of the IG NGS method, the superiority of the IG NGS method for diagnosing and tracking MRD, the existing limitations, future trends, and potential development directions. With the continuing increasing sensitivity and affordability of HTS technology, the routine use of IG NGS-based MRD monitoring in clinical practice is expected within the near future, with robust performance and reasonable per-sample cost.

## Supplementary Information

**Additional file1: Fig. S1** Schematic representation of Ig heavy and light chain rearrangements in normal B cells. (A) Complete VH-D-JH recombination in the IGH locus (14q32). (B) The classical Vκ-Jκ recombination in the IGK locus (2p11) (up), the inversion Vκ(D)-Jκ rearrangement (middle), and Kde-mediated deletions by RSS-intron or Cκ (below). V, variable. D, diversity. J, joining. C, constant.

## Data Availability

All data generated or analyzed during this study are included in this published article [and its supplementary information files].

## Abbreviations

ALL: Acute lymphoblastic leukemia
ASO: Allele-specific oligonucleotide
BCR: B cell antigen receptor
BM: Bone marrow
CAR-T: Chimeric antigen receptor-T cell
CDR3: Complementarity determining regions 3
cfDNA: Cell free DNA
CLL: Chronic lymphocytic leukemia
CR: Clinical remission
COO: Cell of origin
CSR: Class switch recombination
CTC: Circulating tumor cell
ctDNA: Circulating tumor DNA
ddPCR: Digital droplet PCR
DSB: Double strand break
DLBCL: Diffuse large B cell lymphoma
FL: Follicular lymphoma
GC: Germinal center
GCB: Germinal center B cell
HCDR3: Heavy chain CDR3
IG: Immunoglobulin
IGH: Immunoglobulin heavy chain
LCDR3: Light-chain CDR3
MCL: Mantle cell lymphoma
MFC: Multi-color flow cytometry
MM: Multiple myeloma
MRD: Minimal residual disease
NGF: Next-generation flow cytometry
NGS: Next-generation sequencing
N-gly: Asparagine-X-serine/threonine
OS: Overall survival
PC: Plasma cell
PCR: Polymerase Chain Reaction
PET-CT: Positron emission tomography-computed tomography
PFS: Progress free survival
RAG: Recombination activating gene
RSS: Recombination signal sequence
SHM: Somatic hypermutation
V: Variable
