# Supplementary material for: Next-generation sequencing for MRD monitoring in B-lineage malignancies: from bench to bedside
Source: Exp Hematol Oncol. 2022 Sep 3;11:50. doi: 10.1186/s40164-022-00300-2 (PMC9440501; doi:10.1186/s40164-022-00300-2)
Supplement: Supplementary file 1 — Additional file1: Fig. S1 Schematic representation of Ig heavy and light chain rearrangements in normal B cells. (A) Complete VH-D-JH recombination in the IGH locus (14q32). (B) The classical Vκ-Jκ recombination in the IGK locus (2p11) (up), the inversion Vκ(D)-Jκ rearrangement (middle), and Kde-mediated deletions by RSS-intron or Cκ (below). V, variable. D, diversity. J, joining. C, constant. [file 40164_2022_300_MOESM1_ESM.pdf]

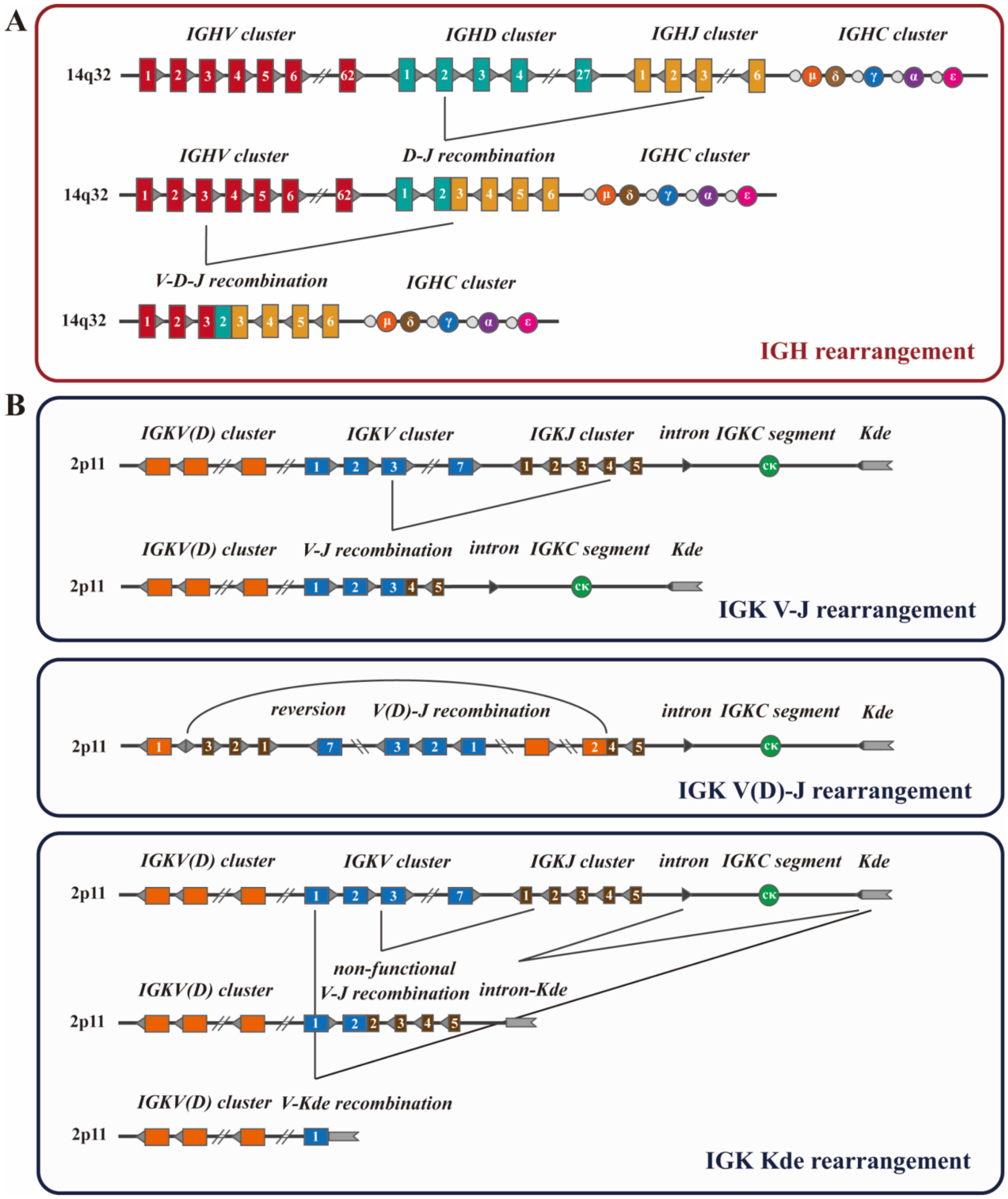

Supplementary Figure 1. Schematic representation of Ig heavy and light chain rearrangements in normal B cells. (A) Complete VH-D-JH recombination in the IGH locus (14q32). **(B)** The classical Vκ-Jκ recombination in the IGK locus (2p11) (up), the inversion Vκ(D)-Jκ rearrangement (middle), and Kde-mediated deletions by RSS-intron or Cκ (below). V, variable. D, diversity. J, joining. C, constant.
